# Duck STING mediates antiviral autophagy directing the interferon signaling pathway to inhibit duck plague virus infection

**DOI:** 10.1186/s13567-024-01338-2

**Published:** 2024-06-28

**Authors:** Bin Tian, Yanming Tian, Xuetong Wang, Dongjie Cai, Liping Wu, Mingshu Wang, Renyong Jia, Shun Chen, Dekang Zhu, Mafeng Liu, Qiao Yang, Ying Wu, Xinxin Zhao, Shaqiu Zhang, Di Sun, Juan Huang, Xumin Ou, Zhen Wu, Anchun Cheng

**Affiliations:** 1 Engineering Research Center of Southwest Animal Disease Prevention and Control Technology, Ministry of Education of the People’s Republic of China, Chengdu, 611130 China; 2 Research Center of Avian Disease, College of Veterinary Medicine, Sichuan Agricultural University, Chengdu, 611130 Sichuan China; 3 Key Laboratory of Animal Disease and Human Health of Sichuan Province, Sichuan Agricultural University, Chengdu, 611130 Sichuan China

**Keywords:** STING, selective autophagy, LC3B, LIR, duck plague virus

## Abstract

Migratory birds are important vectors for virus transmission, how migratory birds recognize viruses and viruses are sustained in birds is still enigmatic. As an animal model for waterfowl among migratory birds, studying and dissecting the antiviral immunity and viral evasion in duck cells may pave a path to deciphering these puzzles. Here, we studied the mechanism of antiviral autophagy mediated by duck STING in DEF cells. The results collaborated that duck STING could significantly enhance LC3B-II/I turnover, LC3B-EGFP puncta formation, and mCherry/EGFP ratio, indicating that duck STING could induce autophagy. The autophagy induced by duck STING is not affected by shRNA knockdown of ATG5 expression, deletion of the C-terminal tail of STING, or TBK1 inhibitor BX795 treatment, indicating that duck STING activated non-classical selective autophagy is independent of interaction with TBK1, TBK1 phosphorylation, and interferon (IFN) signaling. The STING R235A mutant and Sar1A/B kinase mutant abolished duck STING induced autophagy, suggesting binding with cGAMP and COPII complex mediated transport are the critical prerequisite. Duck STING interacted with LC3B through LIR motifs to induce autophagy, the LIR 4/7 motif mutants of duck STING abolished the interaction with LC3B, and neither activated autophagy nor IFN expression, indicating that duck STING associates with LC3B directed autophagy and dictated innate immunity activation. Finally, we found that duck STING mediated autophagy significantly inhibited duck plague virus (DPV) infection via ubiquitously degraded viral proteins. Our study may shed light on one scenario about the control and evasion of diseases transmitted by migratory birds.

## Introduction

The host innate immune response is the first line of defense against the invasion of pathogenic microorganisms. Various pattern recognition receptors (PRR) play an essential role in the specific recognition of pathogen-associated molecular patterns (PAMP) associated with invading pathogens. PAMP, recognized by PPR, initiate an innate immune response, characterized by the induction of interferon (IFN), proinflammatory cytokines, and IFN-stimulated gene (ISG) production [1]. The DNA sensors cGAS (cyclic GMP-AMP (cGAMP) synthase) recognize a variety of cytoplasmic DNA [2]. After binding to cytoplasmic double-stranded DNA, cGAS will be activated and further catalyzes the synthesis of cyclic GMP-AMP (cGAMP) using ATP and GTP [3]. cGAMP serves as a second messenger, binding to interferon gene stimulators (STING, also known as MITA, ERIS, MPYS, or TMEM173) and initiates STING activation, which then recruits tank-binding kinase 1 (TBK1) and interferon regulatory factor 3/7 (IRF3/7) [4]. STING and IRF3/7 are subsequently phosphorylated by TBK1 and IRF3/7 transport to the nucleus for recognition, binding to downstream gene promoters to produce IFN-I, ISG, and cytokines [5].

After binding to cGAMP, oligomerization and conformational structure changes of STING commences immediately, followed by transportation from ER to the ER Golgi intermediate compartment (ERGIC) and trans Golgi network (TGN) [6], where tank-binding kinase 1 (TBK1) is recruited into the network subsequently to phosphorylate STING and IRF3/7 [7, 8]. The transport process of STING is an absolutely necessary early event to activate and mediate the antiviral immune response and autophagy, in which the coat protein complex-II (COPII) adaptor proteins play an important role [9]. The formation of COPII complexes is an orderly process, in which the SAR1 subunit is the most important regulatory molecule. When the target protein cargo arrives at ER, SEC12 initiates the exchange between SAR1-GDP and GTP to form SAR1-GTP; SAR1-GTP is then translocated to ER and recruits the intima proteins of COPII to form SAR1-GTP/SEC23/SEC24 complex [10]. The complex wraps the target protein and then recruits the COPII outer membrane proteins SEC13 and SEC31 to form a complete COPII transport complex. Intact COPII complexes transport target cargo to the Golgi apparatus, autophagosomes, and cell membranes via the ERES (ER exit sites) (14). After binding with cGAMP, STING interacts with SEC24, and then recruits SAR1 to ERES to form the COPII complex and transport STING to ERGIC or the Golgi apparatus [9]. However, knowledge of the function of STING in poultry is poorly known, limiting research and development of many avian transmitted disease prevention and control.

IRF3 is almost absent in birds, and chickens do not have RIG-I [11]. Chicken STING can inhibit the proliferation of avian influenza and Newcastle disease virus in chicken cells by inducing IFNβ production through interaction with MDA5 and DDX3X, and this effect is independent of Rig-I [12, 13]. In addition, chicken DNA virus sensor DDX41 activated the IFN-β signaling pathway by interacting with chicken STING [14]. Avian oncogenic herpesvirus antagonizes the cGAS-STING DNA-sensing pathway to mediate immune evasion [15], implying that the cGAS-STING pathway is working in the chicken. Sequence alignment shows that Duck STING (DuSTING) protein shares 71.1, 43.4, and 33.3% identity with chickens, humans, and zebra fish, respectively. Overexpression of DuSTING in duck embryo fibroblasts (DEF) strongly activated IFN-β promotor activity, and deletion mutant analysis revealed that the first 42 aa containing the first transmembrane (TM) domains and the last 32 aa containing a part of the C-terminal tail (CTT) are essential for its IFN-β activation [16]. Furthermore, goose and duck STING plays an important role in host anti-duck plague virus infection and RNA virus through an IFN-dependent signaling pathway [17, 18], whereas IRF7 may be involved in both STING and MAVS mediated IFN-beta Signaling [19]. In general, studies on avian STING mainly focus on its function in mediating the IFN pathway, while research on autophagy is still missing.

Autophagy is a highly conserved intracellular degradation process in eukaryotic evolution that is required to maintain homeostasis and eliminate damaged organelles, protein aggregates, and invading pathogens [20–22]. STING, as an important adaptor in the innate immune system, has also been proved to have the function of mediating selective autophagy, which is highly conserved and more primitive in Nematostella, Xenopus, Danio rerio, Mus musculus, and Homo sapiens [9]. Previous studies have found that the autophagy-inducing activity of STING pre-dates its interferon-inducing activity during evolution [9]. Autophagy induced by STING has a direct antiviral infection effect, and the process takes a shorter time (30 min to 2 h), which may make up for signaling pathways such as IFN and cytokines that require a longer time to exert antiviral effect, which may be the reason why the STING mediated autophagy function is highly conserved and preserved in different species [23]. STING-mediated autophagy is not only induced by DNA virus infection, but also RNA virus infection activates the RLR-STING-PERK pathway and induces STING-mediated selective autophagy [24], implying that STING-mediated autophagy is very important in the resistance to the early stages of both RNA and DNA virus infection. STING mediated autophagy is highly conserved in animals, but the mechanism of STING mediated antiviral autophagy in birds remains unclear.

Migratory birds are important vectors for the transmission of viruses, such as avian influenza virus, West Nile virus, and duck plague virus (DPV, a member of alphaherpesviridae) [25–30]. Therefore purporting to illustrate how migratory birds resist viruses and viruses evade antiviral innate immunity is pivotal but still enigmatic. As the model animal of waterfowl, deciphering the antiviral immune mechanism of ducks is of vital importance for the control and prevention of migratory bird-transmitted diseases. Our previous study shows that duck STING plays an important role in host anti-duck plague virus infection through an IFN-dependent signaling pathway [17]. However, the precise mechanism by which the aquatic STING regulates autophagy against DPV infection remains unclear. In this study, we found that duck STING is a potential autophagy receptor, directly interacting with LC3 via its LIR motifs to mediate autophagic degradation of viral proteins and tune the innate immune responses.

## Materials and methods

### Ethics statement

The study was approved by the Committee of Experiment Operational Guidelines and Animal Welfare of Sichuan Agricultural University (Approved permit number XF2014-18). All animal experiments were conducted in accordance with the approved guidelines.

### Antibodies, virus strains, plasmids, and agents

The virulent strain CHv used in this study is a virulent DPV strain that was isolated and characterized by our lab. The recombinant virulent strain of DPV, BAC-CHv-EGFP (CHv-GFP), was constructed by our research center. Each of these DPV strain proliferations and tissue culture infectious dose 50 (TCID_50_) titrated detection were performed in DEF cells. Anti-Flag, Myc, HA, GAPDH antibodies were purchased from MBL (Japan). Anti-IgG and Anti-LC3B antibodies were purchased from Cell Signaling Technology (USA). Anti-GST antibody were purchased from Abmart (China). DMXAA, Bafinomycin A1 (BAF A1), MG132, Chloroquine (CQ), and BX795 were purchased from MCE (USA). All the host genes used here were cloned from DEF cells, then according to the manufacturer (Mona, China), the STING wild type, STING mutants (LIR1-7 mutants, Y29A/ L32A, W88A/L91A, Y111A/L114A, Y170A/I173A, W212A/L215A, Y295A/L298A, Y312A/I315A, mutant LIR4/7 Y170A/I173A/ Y312A/I315A, V158M, R235A, S369A, L377A, S379A, and Δ342-382), LC3B-FLAG, LC3B-EGFP, mCherry-EGFP-LC3B, Sar1A, Sar1A H79A, Sar1B, Sar1B H79A, DPV US3-Flag, DPV UL40-Flag, DPV UL49-Flag, DPV UL54-Flag plasmids were generated into the eukaryotic expression vector pcaggs. Duck GST-LC3B and GST-LC3B-F52A/L53A mutant were constructed into the prokaryotic expression vector pGEX-4T-1. The construction of duck IFN-β-luciferase reporter plasmid (pGL-IFN-β-luc) was described previously [17].

### Duck embryo fibroblast isolation, culture, and transfection

Preparation and culture of duck embryo fibroblast (DEF) cells were consistent with our previously established protocol [31]. Briefly, 9-day-old duck embryos were cleaned, and the muscle tissues were washed with PBS, cut and digested with 0.1% trypsin for 8 min. After digestion, single cells were collected and plated in cell culture plates or dishes and cultured in DMEM supplemented with 10% fetal bovine serum (FBS; Gibco-BRL, Carlsbad CA, USA) and 1% penicillin–streptomycin (Beyotime China). Usually, DEF cells were cultured to 100% confluence for 24 h and then passaged and sub-cultured for use in subsequent assays. For plasmids or shRNA transfection into DEF, the Hieff Trans^R^ Liposomal Transfection Reagent (Yeasen, China) was applied following the manufacturer’s instruction.

### shRNA interference

The shRNA used in this study were all designed and cloned into PTSB-U6-PGK-Fluor-2A-ARGs by TsingkeBiotechnology (Beijing, China). Cells were transfected with shRNA expressing plasmids using Hieff Trans^R^ Liposomal Transfection Reagent (Yeasen, China) according to the manufacturer’s instructions. The shRNA sequences used in this study were as follows: shATG5, #1 5′- CACAAAUUUGAUAAGCAAA-3′, #2 GGGAAGCCGAGCCTTACTA, #3 GAATTGTAATATAATATTC; and shSTING, 5′- GCAGGAACCTACAGGCTCATT-3′; and shNC, 5’-GTTCTCCGAACGTGTCACGT-3’.

### GST pulldown assay

GST, GST-LC3B and GST-LC3B-F52A/L53A proteins were expressed and purified from *Escherichia coli* BL21 (DE3). Cell lysates were incubated with prepared GST, GST-LC3B, or GST-LC3B-F52A/L53A protein at 4 °C overnight, and then washed five times with phosphate buffered saline and tween-20 (PBST). The precipitated complex was boiled for 5 min with loading buffer and subjected to SDS-PAGE and immunoblotting.

### Immunoprecipitation and immunoblot analysis

After transfection or viral infection, cells were washed with PBS and lysed with IP lysate supplemented with a protease inhibitor cocktail (Sigma) for 30 min to release proteins, and then centrifuged at 12,000*g* at 4 °C for 10 min. The supernatants were incubated with anti-Flag, Myc or LC3B affinity beads or the indicated antibodies followed by protein A/G agarose beads. The immunoprecipitated proteins were washed with the 1% Triton buffer at least 5 times for 10 min each time. Cell lysates or immunoprecipitates were separated by SDS–PAGE and then transferred to PVDF membrane (Bio-rad). The membranes were sealed with 5% skim milk powder dissolved in PBST for 3 h and then incubated with the appropriate primary and secondary antibodies, and then visualized with the Bio-Rad system (Bio-Rad, Germany).

### Indirect immunofluorescence assay (IFA)

The DEF cells were transfected with the indicated plasmids and then fixed with 4% neutral buffered paraformaldehyde for 20 min, permeabilized with 0.2% Triton X-100 for 10 min, blocked with 10% BSA dissolved in PBS for 30 min at 37 °C, and then incubated with primary antibodies against Myc at 4 °C overnight. The cells were incubated with Alexa Fluor 594–conjugated goat anti-mouse secondary antibodies for 1 h at room temperature. The images were observed using an LSM 510 Zeiss confocal micro scope (Carl Zeiss Jena, Germany).

### Virus infection and determination of TCID_50_

For viral infection, the required dose of virus was diluted DMEM, incubated with the cells at 37 °C for 1 h, then washed twice with PBS, and cultured in the corresponding medium with 2% FBS and 1% penicillin–streptomycin. The culture supernatant of virus-infected cells was collected and the TCID_50_ of DEF was determined by 10 times continuous dilution.

### Dual-luciferase reporter assays

The duck IFN-β-luciferase reporter plasmid (pGL-IFN-β-luc), and pRL-TK, were co-transfected into DEF cells together with the indicated plasmids for 36 h. The Dual-Luciferase Reporter Assay System was purchased from Promega. The cells were collected and lysed. Subsequently, IFN-β reporter gene activity was detected using the dual-luciferase reporter assay system according to the manufacturer’s protocols. Data were normalized based on the Renilla luciferase activity.

### Statistical analysis

Data are expressed as the mean and standard error of the mean (SEM), and the significance of differences between groups was evaluated using the Student’s *t* test or one-way analysis of variance followed by Tukey post hoc test. Asterisks indicate the level of statistical significance (*P < 0.05; **P < 0.01; ***P < 0.001). Graphs were plotted and analyzed using GraphPad Prism software, version 6.0 (GraphPad Software, La Jolla, CA, USA).

## Results

### Duck STING can induce autophagy

**Figure 1:**
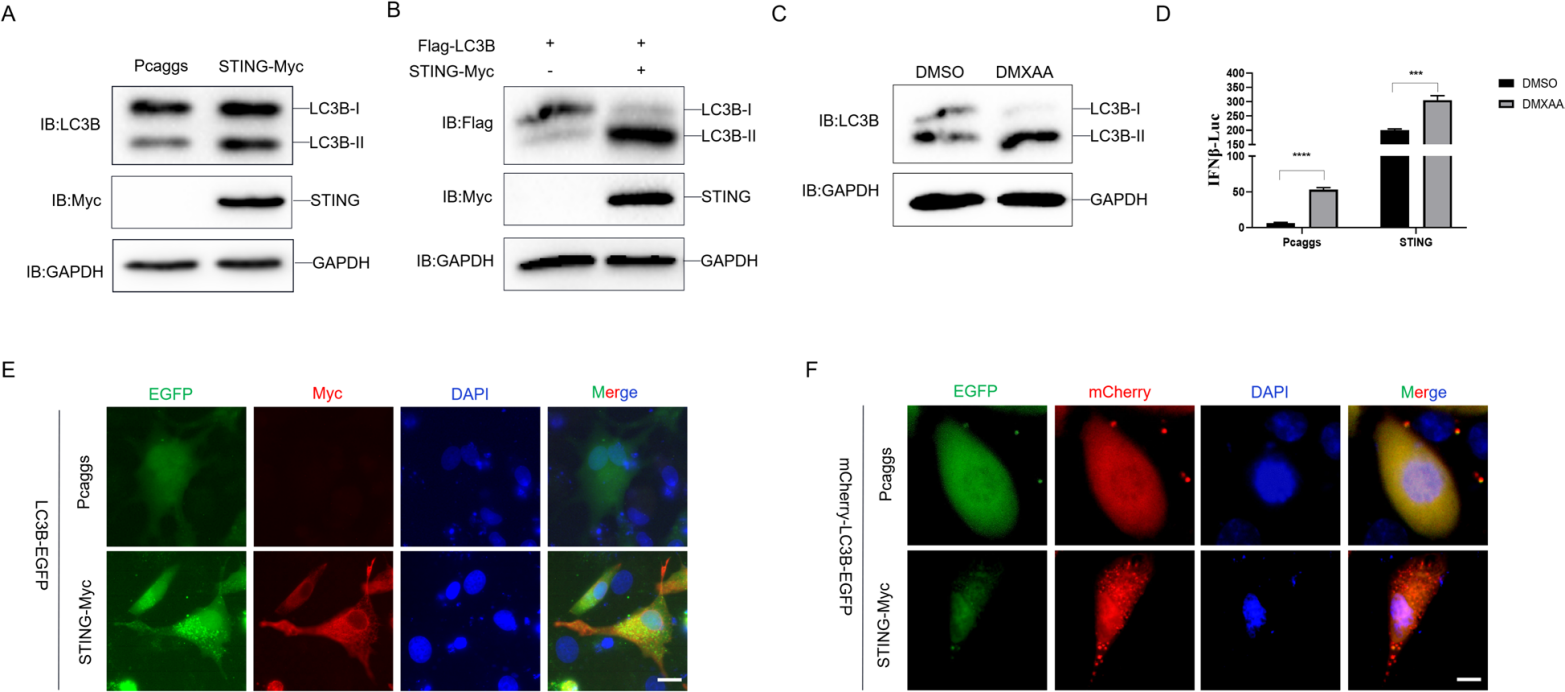
**Duck STING can induce autophagy.** **A** The STING-Myc expressed plasmid was transfected into DEF, the level of STING, LC3B, and GAPDH were detected through immunoblotting at 36 h post-transfection. **B** The Flag-LC3B expressing plasmids were transfected into DEF together with or without STING-Myc expressing plasmid, the level of STING, LC3B, and GAPDH were detected through immunoblotting at 36 h post-transfection. **C** DEF were treated with STING agonists, DXMAA, for 12 h, and then the LC3B, and GAPDH were detected through immunoblotting. **D** The DEF cells were transfected with phRL-TK Renilla luciferase plasmid, luciferase reporter genes, together with or without STING expressing plasmid for 24 h, then the cells were treated with DMXAA or DMSO for 12 h, the luciferase activity was measured and normalized to renilla luciferase activity. **E** The LC3B-EGFP expressing plasmid was transfected into DEF together with or without STING-Myc expressing plasmid, the EGFP expression was photographed and collected using a fluorescence microscope. The scale bar is 20 µm. **F** The mCherry-EGFP-LC3B expressing plasmid was transfected into DEF together with or without STING-Myc expressing plasmid, the EGFP and mCherry expression was photographed and collected using a fluorescence microscope. The scale bar is 20 µm.

Our previous study has proved that the duck STING is able to inhibit the duck plague virus (DPV) infection in DEF cells through an IFN-dependent signaling pathway [17]. However, recent studies have found that autophagy induction via STING trafficking is a primordial function of the cGAS pathway to directly tune the innate immune response [9, 32]. Hereafter, we attempt to investigate whether duck STING is able to induce antiviral autophagy. Firstly, we overexpressed duck STING in DEF cells and found that the endogenous duck LC3BI/LC3BII conversion was obviously enhanced (Figure 1A). For convenience detection of duck LC3B in DEF cells, we constructed the duck Flag-LC3B expressed plasmid (Flag tag was fused to the N terminal of duck LC3B) and expressed it together with duck STING. We also observed the enhanced LC3BI/LC3BII conversion by duck STING overexpression (Figure 1B). We then tested the STING agonist, DMXAA, and observed that DMXAA enhanced the endogenous duck LC3BI/LC3BII conversion (Figure 1C). Figure 1D shows that DMXAA is suitable for DEF cells, indicating that the endogenous duck STING activation is able to induce autophagy in duck cells. In order to visualize the autophagy occurring in duck cells, we constructed the duck LC3B-EGFP expressing plasmid, and co-transfected it with duck STING-Myc expressing plasmid in DEF cells. We saw that duck STING significantly increased puncta of duck LC3B-EGFP (Figure 1D). Furthermore, in order to visualize autolysosome localized duck LC3B, we constructed the mCherry-LC3B-EGFP expressing plasmid with duck LC3B, in which the mCherry is stable but the EGFP is sensitive to an acidic environment, being degraded in the lysosome acidic environment. After co-expression of mCherry-LC3B-EGFP with duck STING in DEF cells, we found that the duck STING obviously enhanced the mCherry/EGFP ratios, indicating that duck LC3B located into the lysosome after STING stimulation (Figure 1E). Taken together, these data demonstrate that the duck STING is able to induce autophagy in duck cells.

### Duck STING induced autophagy does not depend on TBK1 and ATG5

**Figure 2:**
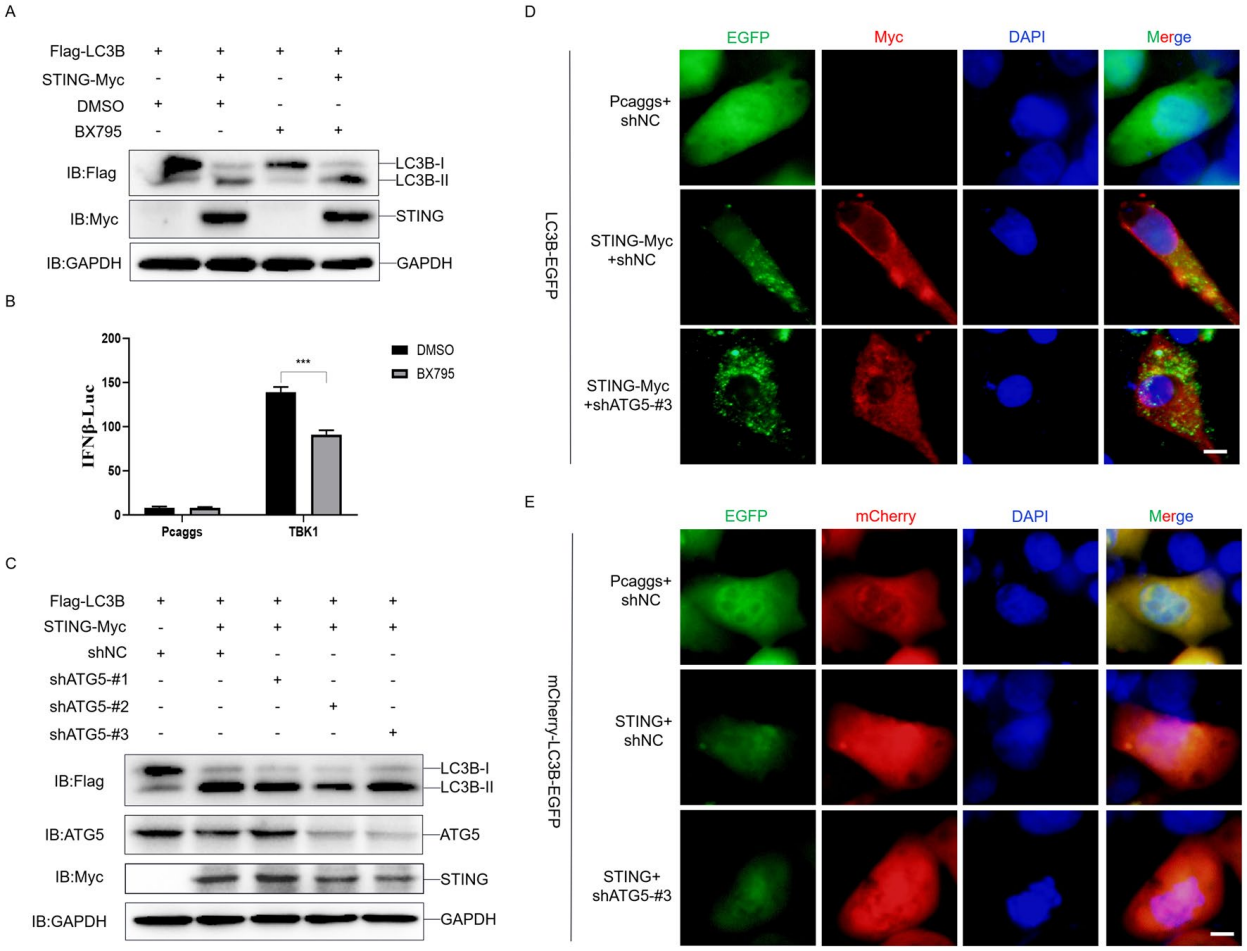
**Duck STING induced autophagy does not depend on TBK1 and ATG5.** **A** The Flag-LC3B expressing plasmids was transfected into DEF together with or without STING-Myc expressing plasmid for 24 h, then the DEF were treated with the TBK1 inhibitor, BX795 for 12 h, and the level of STING, LC3B, and GAPDH were detected through immunoblotting assays. **B** The DEF cells were transfected with phRL-TK Renilla luciferase plasmid, luciferase reporter genes, together with or without TBK1 expressing plasmid for 24 h, then the cells were treated with BX795 or DMSO for 12 h, the luciferase activity was measured and normalized to renilla luciferase activity. **C** The LC3B-Flag expressing plasmids were transfected into DEF together with or without STING-Myc expressing plasmid, and with shNC or different shRNA targeting ATG5 (#1, 2, and 3), and the level of STING, LC3B, ATG5 and GAPDH were detected through immunoblotting at 36 h post-transfection. **D** The LC3B-EGFP expressing plasmid was transfected into DEF together with or without STING-Myc, shNC, or shATG5-#3 expressing plasmid, the EGFP expression was photographed and collected using a fluorescence microscope. The scale bar is 20 µm. **E** The mCherry-EGFP-LC3B expressing plasmid was transfected into DEF together with or without STING-Myc, shNC, or shATG5-#3 expressing plasmid, the EGFP and mCherry expression was photographed and collected using a fluorescence microscope. The scale bar is 20 µm.

We further evaluated whether duck STING induced autophagy needs the TBK1 activity or the typical autophagy pathway. First, we treated the STING expressing DEF cells with the TBK1 inhibitor, BX795, and measured the duck LC3BI/LC3BII conversion. We observed that BX795 did not change the STING induced LC3BI/LC3BII conversion (Figure 2A). Figure 2B shows that BX795 is also suitable for DEF cells, indicating that the TBK1 is dispensable for duck STING induced autophagy. Furthermore, we knocked down duck ATG5 in the STING expressing DEF cells and the shATG5#3 knockdown efficacy was most significant (Figure 2C). We then checked the expression of LC3BI/LC3BII, and found that knockdown of ATG5 had no impact on the duck STING induced LC3BI/LC3BII conversion (Figure 2C). Moreover, the knockdown of duck ATG5 neither changed the puncta of duck LC3B-EGFP nor the ratios of mCherry/EGFP stimulated by duck STING (Figures 2D and E), implying ATG5 is not necessary for duck STING induced autophagy. These data indicate that duck STING induced autophagy is not dependent on TBK1 kinase activity and the typical autophagy pathway.

### The C-terminal tail of STING is dispensable for autophagy induction

**Figure 3:**
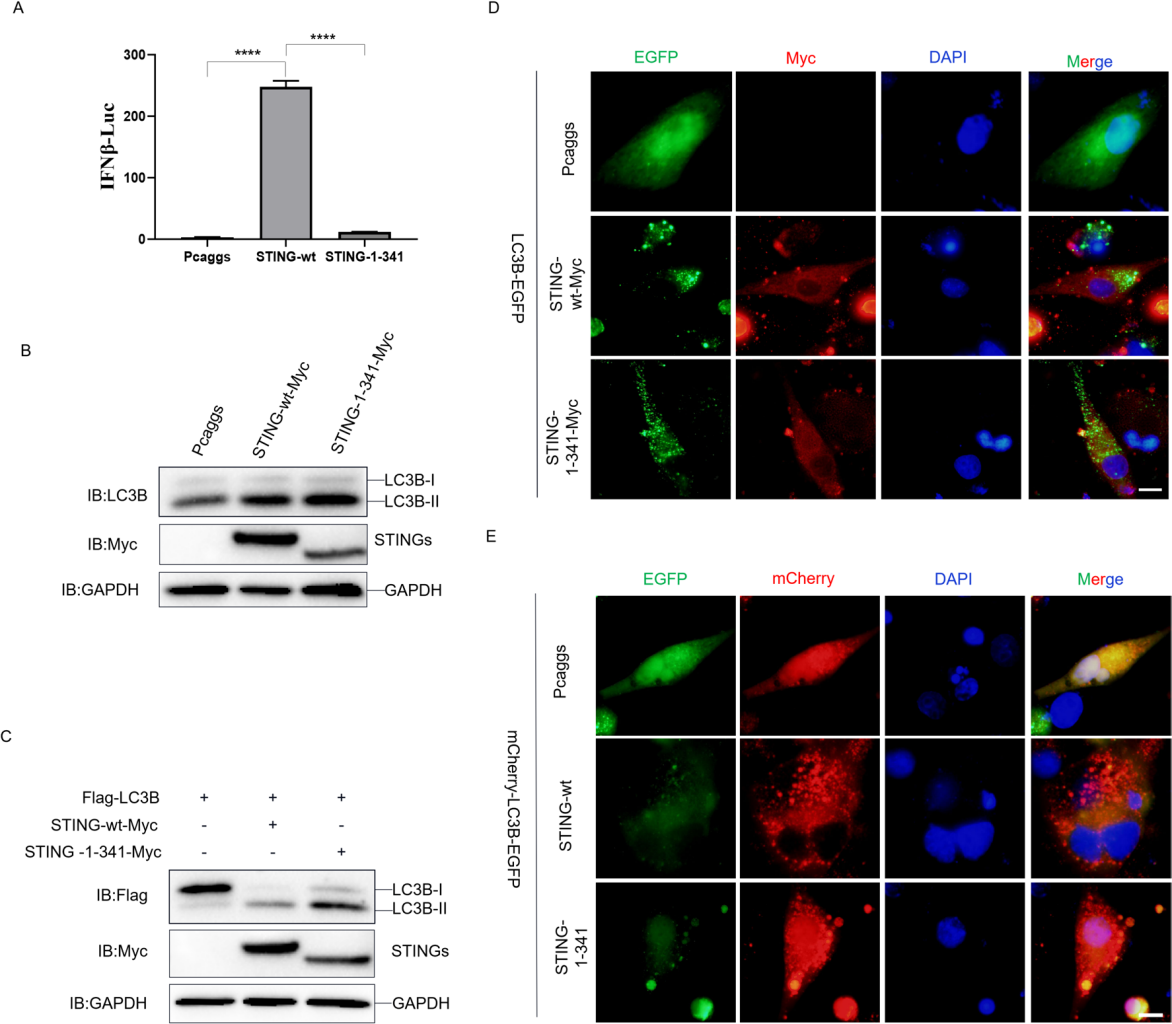
**The C terminal of duck STING is dispensable for autophagy induction.** **A** The DEF cells were transfected with phRL-TK Renilla luciferase plasmid, luciferase reporter genes, together with STING-wt or STING-1–341 expressing plasmid, respectively. At 36 h post-transfection, the luciferase activity was measured and normalized to renilla luciferase activity. **B** The STING-wt-Myc and STING-1–341-Myc expressed plasmid was transfected into DEF, the level of STING, LC3B, and GAPDH were detected through immunoblotting at 36 h post-transfection. **C** The LC3B-Flag expressing plasmids were transfected into DEF together with or without STING-wt-Myc and STING-1–341-Myc expressed plasmid, and the level of STING, LC3B, and GAPDH were detected through immunoblotting at 36 h post-transfection. **D** The LC3B-EGFP expressing plasmid was transfected into DEF together with or without STING-wt-Myc and STING-1–341-Myc expressed plasmid, the EGFP expression was photographed and collected using a fluorescence microscope. The scale bar is 20 µm. **E** The mCherry-EGFP-LC3B expressing plasmid was transfected into DEF together with or without STING-wt-Myc and STING-1–341-Myc expressed plasmid, the EGFP and mCherry expression was photographed and collected using a fluorescence microscope. The scale bar is 20 µm.

The C-terminal tail (CTT) of STING is critical for IFN-I and ISG production by recruiting TBK1 and activate downstream signaling pathways. Hence, we constructed the CTT deletion truncation of STING (STING-1–341) expressing plasmid to investigate whether the innate immunity mediation function of duck STING could affect the autophagy induction. First, we transfected this duck STING truncation into DEF cells with the firefly luciferase reporter harboring the duck IFN-beta promotor. We found that the duck STING wt obviously stimulated the IFN expression, however, the CTT truncation lost such an ability (Figure 3A), indicating that the CTT of duck STING is indispensable for innate immune responses. Then we expressed the STING wt and STING-1–341 in DEF cells and observed that both of them increased the endogenous and exogenous LC3BI/LC3BII conversion (Figures 3B and C). Furthermore, both STING wt and STING-1–341 induced puncta of duck LC3B-EGFP and increased the ratios of mCherry/EGFP (Figures 3D and E). Together these data suggest that the innate immunity mediation function of duck STING is dispensable for autophagy induction.

### Duck STING associates with duck LC3B

**Figure 4:**
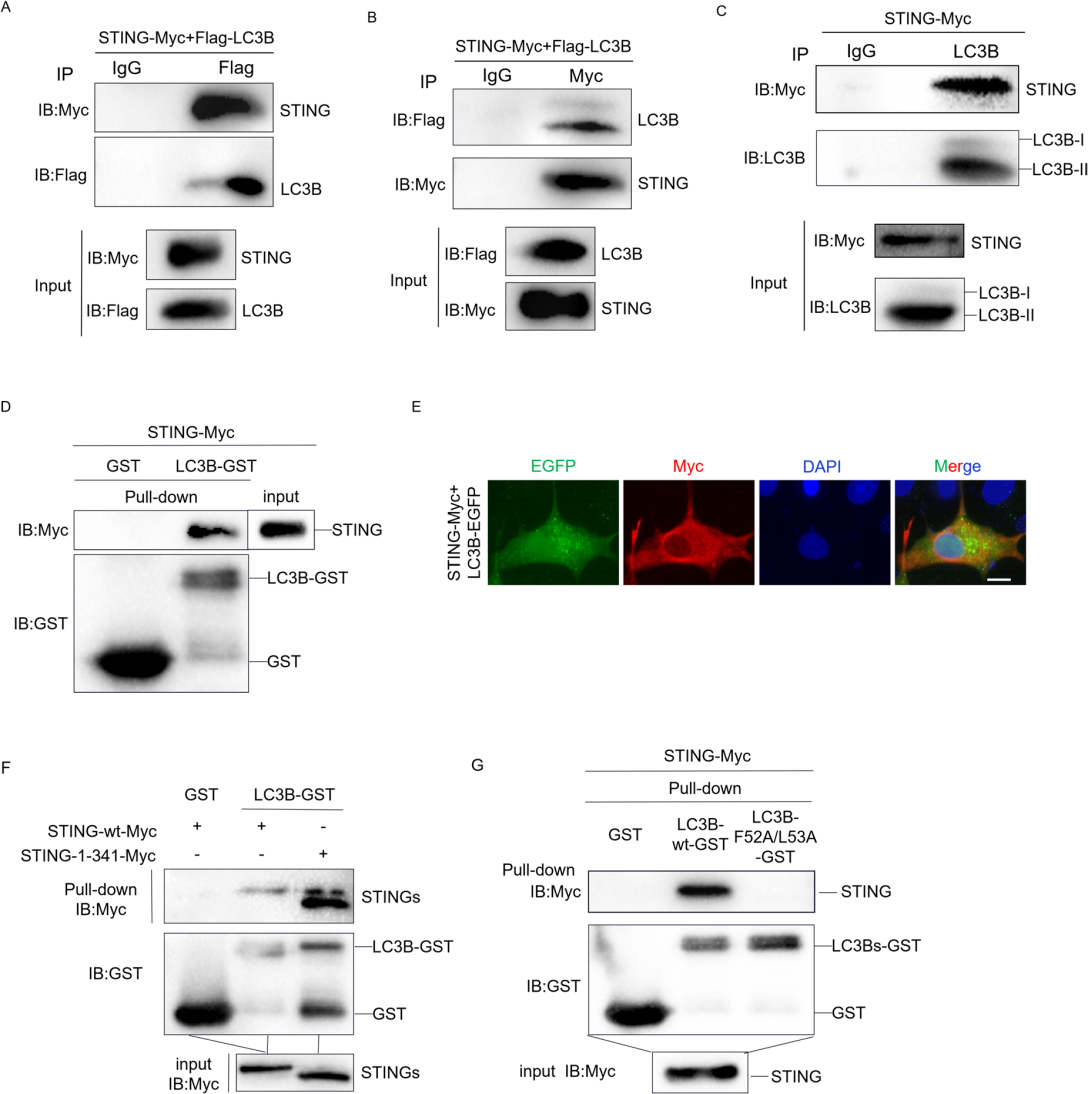
**Duck STING associates with duck LC3B.** **A** and **B** The DEF cells were transfected with STING-Myc and Flag-LC3B expressing plasmids for 36 h, the WCL were used for Co-IP with anti-Flag antibody (**A**) or anti-Myc antibody (**B**) and immunoblot assays. **C** The DEF cells were transfected with STING-Myc expressing plasmids for 36 h, the WCL were used for Co-IP with anti-LC3B antibody and immunoblot assays. **D** Purified GST or LC3B-GST proteins were incubated with lysates of HEK293T cells transfected with STING-Myc expressing plasmid at 4 °C overnight. Immunoblot analysis was then carried out to detect the interaction between duck STING and duck LC3B. **E** The LC3B-EGFP expressing plasmid was transfected into DEF together with STING-Myc expressing plasmid, the LC3-EGFP and STING-Myc expression was photographed and collected using a fluorescence microscope. The scale bar is 20 µm. **F** Purified GST or LC3B-GST proteins were incubated with lysates of HEK293T cells transfected with STING-wt-Myc or STING-1–341-Myc expressing plasmid at 4 °C overnight. Immunoblot analysis was then carried out to detect the interaction between LC3 and STING-wt or STING-1–341. **G** Purified GST, LC3B-wt-GST or LC3B-F52A/L53A-GST proteins were incubated with lysates of HEK293T cells transfected with STING-Myc expressing plasmid at 4 °C overnight. Immunoblot analysis was then carried out to detect the interaction between STING and LC3B-wt or LC3B-F52A/L53A.

Next, we investigated whether duck STING associates with duck LC3B for autophagy induction. Hence, we executed the co-immunoprecipitation (Co-IP) assays to clarify their relationship, which revealed that both Flag-tagged LC3B and endogenous LC3B were bound to ectopically expressed duck STING (Figures 4A, B and C). Furthermore, we prokaryotic expressed and purified the duck LC3B-GST, and then incubated it with the eukaryote expressed duck STING from HEK293T cells for a GST pulldown assay. Then the immunoblotting assay was applied and we found that the duck STING was pulled down with duck LC3B-GST (Figure 4D), but not the GST control, which means duck STING is able to associate with duck LC3B. Then we observed that duck STING is co-localized with duck LC3B-EGFP in DEF cells examined through immune-fluorescence assays (Figure 4E). Furthermore, we found that the CTT deletion of STING (STING-1–341) is still able to associate with duck LC3B-GST dose like the duck STING wt (Figure 4F). More importantly, we found that the duck STING could not associate with duck LC3B LDS mutant (duck LC3B F52A/F53A) any more (Figure 4G), in which the LIR docking site (LDS) of duck LC3B was mutated. This result suggests that duck STING is associated with the LDS of duck LC3B via its LIR.

### The LIR4 and LIR7 of duck STING is critical to interacting with LC3B and inducing autophagy and innate immunity

**Figure 5:**
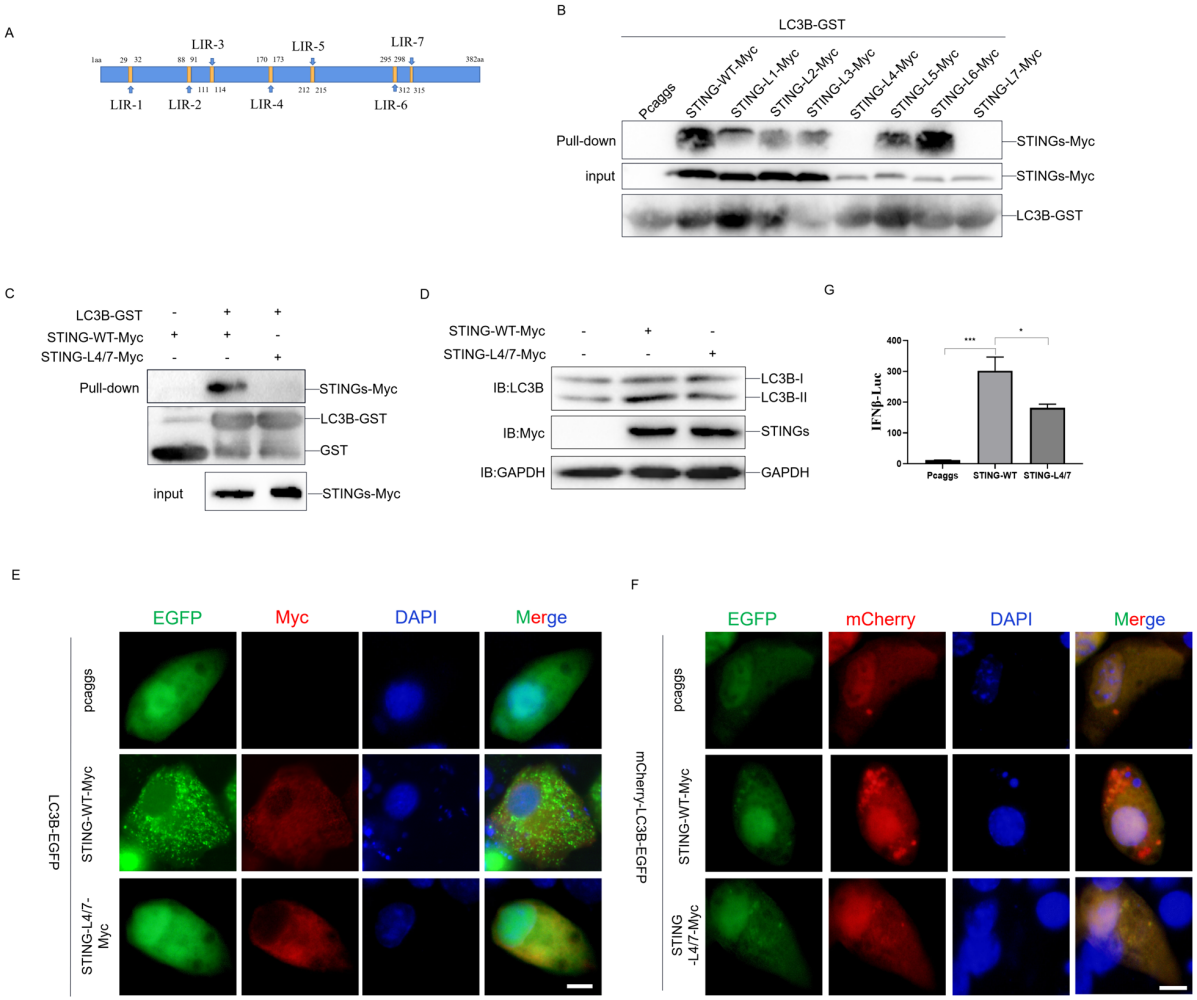
**The LIR4 and LIR7 of duck STING is critical to interact with LC3B and induce autophagy and innate immunity.** **A** Graphical representation of the duck STING protein showing seven potential LIR, the amino acid sequence of the predicted LIR is indicated. **B** Purified LC3B-GST proteins were incubated with lysates of HEK293T cells transfected with STING-WT-Myc or STING-L1–L7-Myc mutant expressing plasmid at 4 °C overnight. Immunoblot analysis was then carried out to detect the interaction between LC3 and STING-WT or -LIR mutants. **C** Purified LC3B-GST proteins were incubated with lysates of HEK293T cells transfected with STING-WT-Myc or STING-L4/7-Myc mutant expressing plasmid at 4 °C overnight. Immunoblot analysis was then carried out to detect the interaction between LC3 and STING-WT or STING-L4/7 mutant. **D** The STING-WT-Myc or STING-L4/7-Myc expressing plasmid was transfected into DEF, the level of STING, LC3B, and GAPDH were detected through immunoblotting at 36 h post-transfection. **E** The LC3B-EGFP expressing plasmid was transfected into DEF together with STING-WT-Myc or STING-L4/7-Myc expressing plasmid, the EGFP expression was photographed and collected using a fluorescence microscope. The scale bar is 20 µm. **F** The mCherry-EGFP-LC3B expressing plasmid was transfected into DEF together with STING-WT or STING-L4/7 expressing plasmid, the EGFP and mCherry expression was photographed and collected using a fluorescence microscope. The scale bar is 20 µm. **G** The DEF cells were transfected with phRL-TK Renilla luciferase plasmid, luciferase reporter genes, together with STING-WT or STING-L4/7 expressing plasmid, respectively. At 36 h post-transfection, the luciferase activity was measured and normalized to renilla luciferase activity.

The above data prompted us to dissect how the LC3B interacting regions (LIR) of duck STING associates with duck LC3B. Thereafter, We predicted the LIR of duck STING using the LC3B Interaction Domain (LIR) prediction database [33] and found 7 LIR may exist in the duck STING (Figure 5A). Then, we constructed each LIR mutant of duck STING (STING-L1–L7) expressing plasmid and executed the LC3B-GST pulldown assays. The immunoblotting data show that the sole LIR4 and LIR7 mutant of STING abolished the interaction with LC3B (Figure 5B). We further constructed the LIR4 and LIR7 double mutant of STING (STING-L4/7) expressing plasmid and operated the LC3B-GST pulldown assays again, and observed that the double LIR mutant of STING could not interact with LC3B anymore (Figure 5C). Consistently, the double LIR4 and LIR7 mutant of STING (STING-L4/7) neither increased the LC3BI/LC3BII conversion (Figure 5D), nor induced LC3B puncta formation (Figure 5E) or enhanced the ratios of mCherry/EGFP (Figure 5F), as compared to STING wt, which substantially promised us that the LIR4 and LIR7 are the key regions for duck STING to induce autophagy. More intriguingly, we transfected this double LIR mutant of duck STING into the DEF cells with the firefly luciferase reporter harboring the duck IFN-β promotor, and found that such a mutant lost the ability to stimulate IFN expression (Figure 5G), implying that STING interaction with LC3B and mediated autophagy may affect the production of innate immunity. These data indicate that the duck STING interacts with LC3B via LIR4 and LIR7 to induce autophagy and tune innate immunity.

### The COPII complex mediated ER exit of STING is critical for STING mediated autophagy

**Figure 6:**
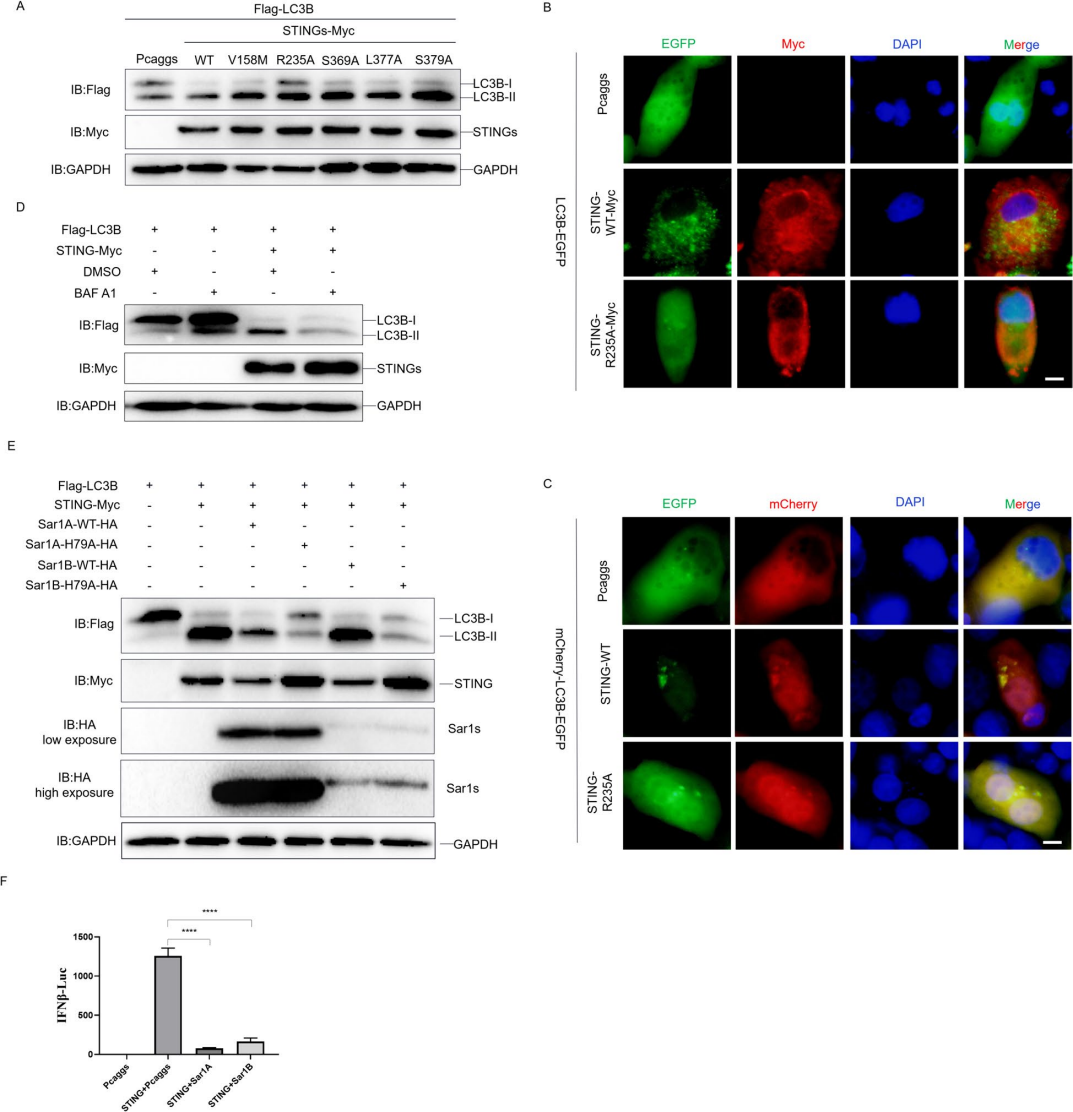
**The COPII complex mediated ER exit of STING is critical for STING mediated autophagy.** **A** The Flag-LC3B expressing plasmids was transfected into DEF together with STING-WT or mutant expressing plasmid, the level of STING, LC3B, and GAPDH were detected through immunoblotting at 36 h post-transfection. **B** The LC3B-EGFP expressing plasmid was transfected into DEF together with STING-WT-Myc or STING-R235A-Myc expressing plasmid, the EGFP expression was photographed and collected using a fluorescence microscope. The scale bar is 20 µm. **C** The mCherry-EGFP-LC3B expressing plasmid was transfected into DEF together with STING-WT or STING-R235A expressing plasmid, the EGFP and mCherry expression was photographed and collected using a fluorescence microscope. The scale bar is 20 µm. **D** The Flag-LC3B expressing plasmids was transfected into DEFs together with or without STING-Myc expressing plasmid for 24 h, then treated with BAF A1 for 12 h, the level of STING, LC3B, and GAPDH were detected through immunoblotting analysis. **E** The DEF cells were transfected with Flag-LC3B, STING-Myc, Sar1A/B-WT or its kinase dead mutant (Sar1A/B-H79A) expressing plasmids, the level of STING, LC3B, Sar1s and GAPDH were detected through immunoblotting at 36 h post-transfection. **F** The DEF cells were transfected with phRL-TK Renilla luciferase plasmid, luciferase reporter genes, STING-WT, together with Sar1A or Sar1B expressing plasmid, respectively. At 36 h post-transfection, luciferase activity was measured and normalized to renilla luciferase activity.

In order to further clarify which phase of the duck STING is involved in autophagy induction, we constructed a series of STING mutants, including, STING-V158M (homologous to human STING V155, enhancing homodimerization), -R235A (homologous to human STING R232, abolishing binding to cGAMP), -S369A (homologous to human STING S366, inhibition phosphorylated by TBK1), -L377A (homologous to human STING L374, blocking the interaction with TBK1), and -S379A (nonsense mutant). We expressed them in DEF cells and found all of these STING mutants induced LC3BI/LC3BII conversion, similarly to that of STING wt, except for the mutant STING-R235A (Figure 6A), suggesting that duck STING induced autophagy is dependent on the cGAMP binding, but is not affected by dimerization formation, interaction with TBK1, or phosphorylation by TBK1. We further show that the STING-R235A mutant neither induced LC3B puncta formation (Figure 6B) nor enhanced the ratios of mCherry/EGFP (Figure 6C) compared to STING wt. Then we expressed the duck STING wt in the DEF cells and treated it with Bafinomycin A1 (BAF A1) to block the organelle membrane fusion and the cargo traffic between organelles. We found that BAF A1 completely terminated the duck STING induced LC3BI/LC3BII conversion (Figure 6D), indicating that STING traffic is important for duck STING mediating autophagy. In the phase of STING activation, after binding with cGAMP, the ER residing STING need to be transported from ER to ERGIC, trans golgi apparatus, endosome, or lysosomes, in which the ER exit is mediated by the COPII complex [9, 34]. For the COPII complex, the kinase activity of SAR1 is a key rate-limiting regulator for cargo traffic [35]. We constructed the duck Sar1A and Sar1B, and their kinase dead mutant, Sar1A-H79A and Sar1B-H79A, expressing plasmids to resolve the role of COPII complex in the duck STING induced autophagy. When Sar1A wt and Sar1B wt expressed with duck STING in DEF cells, the LC3BI/LC3BII conversion ratios was further enhanced by both of them (Figure 6E), however, their kinase dead mutant, Sar1A-H79A and Sar1B-H79A, lost such a capacity as the Sar1 wt did, but phenocopied as the BAF A1 treatment (Figure 6D). Besides, we observed that the level of STING is reduced in the case of Sar1A wt and Sar1B wt overexpression (Figure 6E), implying that over-activation of the COPII complex may bias the STING mediated antiviral activity. As we inferred, overexpression of Sar1A wt and Sar1B wt significantly abrogated the STING mediated IFN (Figure 6F). Taken together, cGAMP binding and COPII complex mediated ER exit are involved in duSTING-mediated autophagy.

### Duck STING induces antiviral autophagy via degrading viral proteins

**Figure 7:**
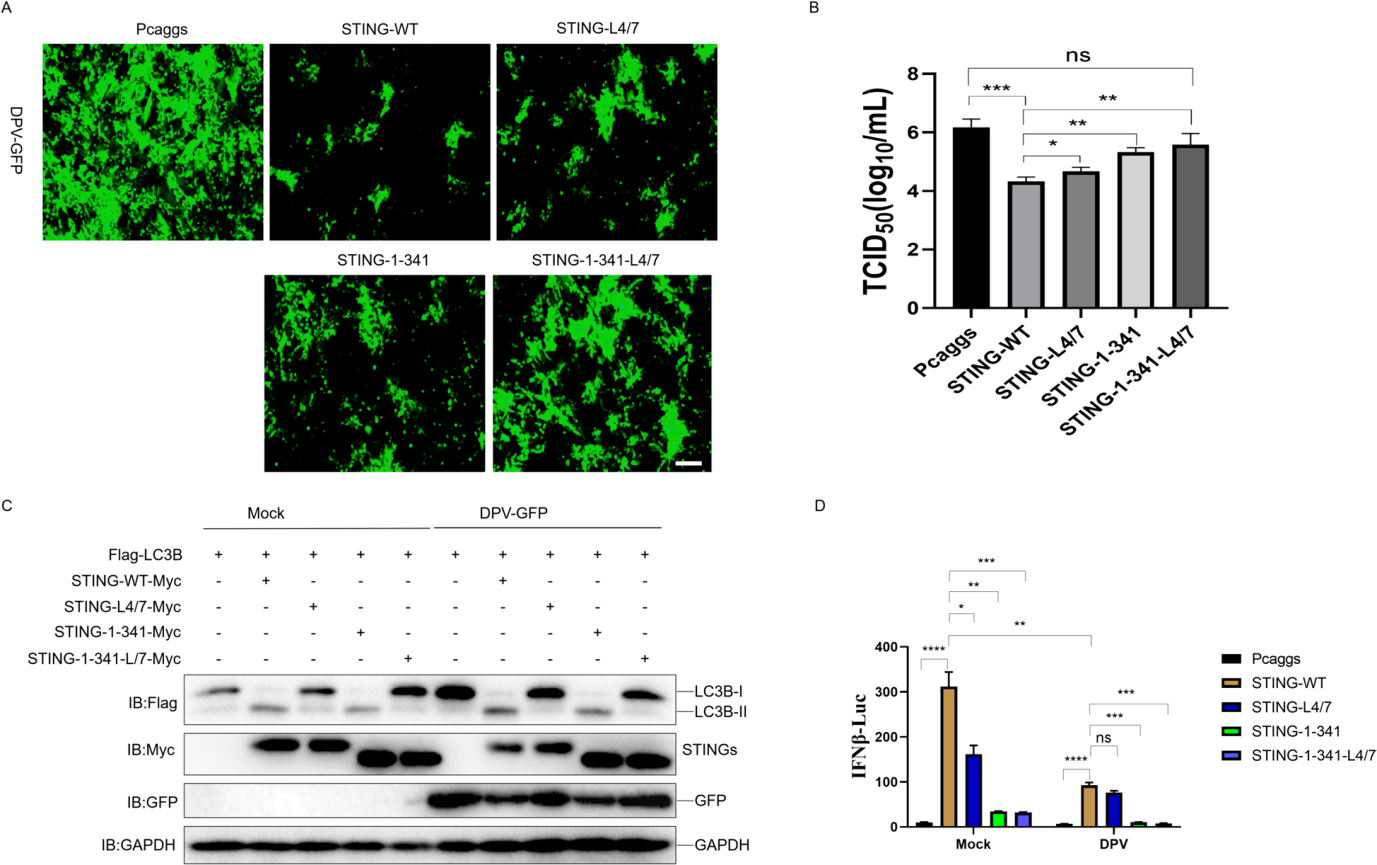
**Duck STING mediated autophagy resists DPV proliferation in DEF.** **A** and **B** The duck STING-WT, STING-LIR 4/7 mutant, STING-1–341 truncation, or STING-1–341-LIR 4/7 expressing plasmids were transfected into DEF cells for 24 h, and infected the cells with 1 MOI of DPV-GFP strain for 36 h, the viral plague of DPV-GFP was collected using a fluorescence microscope. The scale bar is 100 nm (**A**). The cell culture supernatant was collected and the TCID_50_ was determined on DEF cells (**B**). **C** The DEF cells were transfected with Flag-LC3B and STING-WT-Myc, STING-1–341-Myc, STING-L4/7-Myc or STING-1–341-L4/7 expression plasmids for 24 h, followed by infection with DPV-GFP of 1 MOI for another 24 h; the level of STING, LC3B, GFP and GAPDH were detected through immunoblotting. **D** The DEF cells were transfected with phRL-TK Renilla luciferase plasmid, luciferase reporter genes, together with STING-WT or STING-L4/7, STING-1–341, STING-1–341-L4/7 expressing plasmid, at 24 h post-transfection, the DEF were infected with or without DPV-GFP of 1MOI for 24 h; the luciferase activity was measured and normalized to renilla luciferase activity.

**Figure 8:**
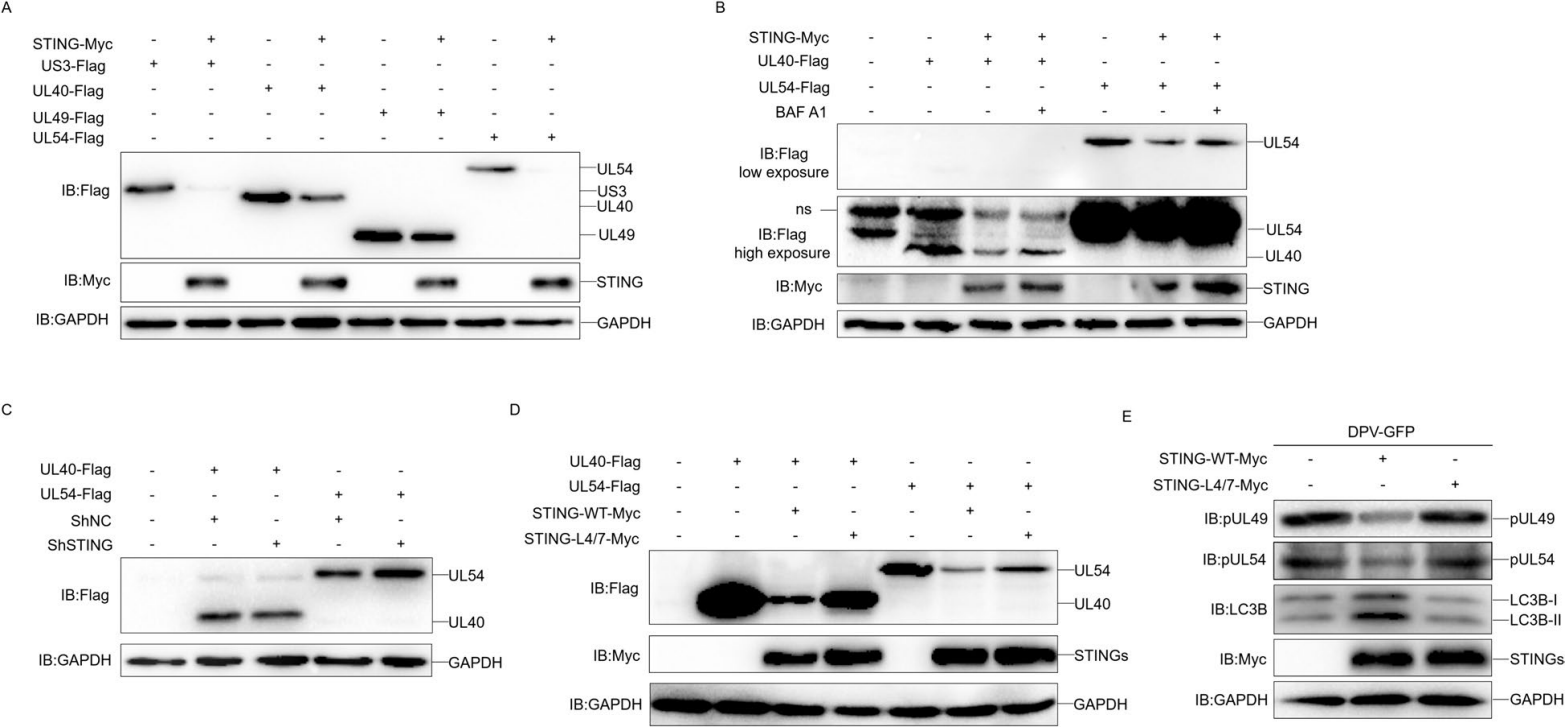
**Duck STING mediated autophagy can ubiquitously degrade viral proteins.** **A** The US3-Flag, UL40-Flag, UL49-Flag or UL54-Flag expressing plasmid was transfected into DEF cells together with or without STING-Myc expressing plasmid for 36 h, the level of US3, UL40, UL49, UL54, STING and GAPDH were detected through immunoblotting analysis. **B** The UL40-Flag or UL54-Flag expressing plasmid was transfected into DEF cells together with or without STING-Myc expressing plasmid for 24 h, then treated with BAF A1 for 12 h, the level of UL40, UL54, STING and GAPDH were detected through immunoblotting analysis. **C** The UL40-Flag or UL54-Flag expressing plasmid was transfected into DEF cells together with shNC or shRNA targeting STING, and the level of UL54, UL40 and GAPDH were detected through immunoblotting at 36 h post-transfection. **D** The UL40-Flag or UL54-Flag expressing plasmid was transfected into DEF cells together with STING-WT-Myc or STING-L4/7-Myc expressing plasmid, and the level of UL54, UL40 and GAPDH were detected through immunoblotting at 36 h post-transfection. **E** The DEF cells were transfected with STING-WT-Myc or STING-1–341-L4/7 expression plasmids for 24 h, then infected with DPV-GFP for another 24 h, the level of pUL49, PUL54, STING, LC3B, and GAPDH were detected through immunoblotting.

We prompted to know whether the duck STING induced autophagy inhibit DPV infection. When we expressed the duck STING-wt, STING-LIR 4/7 mutant, STING-1–341 truncation, and STING-1–341-LIR 4/7 in the DEF cells, respectively, and infected the cells with 1 MOI of DPV-GFP strain, we observed that the DPV replication was obviously inhibited by STING-wt, however, the inhibition gradually attenuated from LIR 4/7 mutant, STING-1–341 truncation, to STING-1–341-LIR 4/7 (Figure 7A). The viral titer in the cell culture supernatant was severely suppressed by duck STING-wt, but gradually increased from LIR 4/7 mutant, STING-1–341 truncation, to STING-1–341-LIR 4/7 (Figure 7B). To explore the effects of STING-WT and its mutants on autophagy and IFN-β-Luc under viral infection conditions, we overexpressed these plasmids in DEF and infected them with DPV-GFP. The results show that STING-WT and STING-1–341, but not STING-LIR 4/7 or STING-1–341-LIR 4/7, induced autophagy under viral infection (Figure 7C). In addition, it is worth noting that DPV infection significantly inhibited STING-WT and mutant mediated IFNβ-Luc activation, under viral infection conditions, STING-1–341 and STING-1–341-LIR 4/7 significantly inhibited IFNβ activation compared to STING-WT (Figure 7D). These data indicate that both autophagy and innate immunity mediated by duck STING is indispensable for antiviral infection against DPV. We then examined whether duck STING autophagy affects viral protein expression, as we detected and found that duck STING expression significantly reduced the protein levels of DPV pUS3, pUL40, pUL49, and pUL54 (Figure 8A), in which the expression of pUL40 and pUL54 was recovered after BAF A1 treatment (Figure 8B). Besides, on the contrary to the STING wt, the STING-LIR 4/7 mutant lost the ability to degrade DPV pUL40 and pUL54 (Figure 8D). Furthermore, the shRNA knockdown of duck STING expression obviously enhanced pUL54 expression level (Figure 8C). To explore the effects of STING-WT and L4/7 on the expression of viral proteins under the condition of viral infection, we overexpressed STING-WT-Myc or STING-LIR 4/7-Myc plasmid in DEF and infected them with DPV-GFP. The results indicate that STING-WT, instead of 4/7, promoted the degradation of pUL49 and pUL54 (Figure 8E). These data suggest that duck STING mediated antiviral autophagy is able to inhibit DPV infection by ubiquitously degrading the viral proteins.

## Discussion

In this study, we reveal the mechanism of duck STING mediated antiviral autophagy. The data demonstrate that duck STING could significantly enhance LC3B-II/I turnover, LC3B-EGFP puncta formation, and mCherry/EGFP ratio, indicating that duck STING could induce autophagy. The autophagy induced by duck STING is not dependent on ATG5, indicating that duck STING activates non-classical selective autophagy. The autophagy activated by duck STING also did not depend on the C-terminal tail or the activity of TBK1, indicating that duck STING mediated autophagy is independent of interaction with TBK1, TBK1 phosphorylation, and innate immunity. Duck STING interacted with the LDS of LC3B through LIR motifs, and their interaction is a decisive factor for autophagy. The binding with cGAMP, activation, and COPII complex mediated transport of duck STING are critical for autophagy induction. The LIR motif mutants of duck STING abolished the interaction with LC3B, hence neither activate autophagy nor IFN expression, indicating that the autophagy activation of duck STING directly affects the function of mediating innate immunity. Finally, we found that duck STING mediated antiviral autophagy function inhibited DPV infection via directly degrading viral proteins.

The destination and disposal of activated STING is very vital for cell homeostasis and survival. If the activated STING is not removed in time, the continuous activation of innate immunity and the continuous production of cytokines will cause metabolic chaos, cell inflammation, even death. After binding with cGAMP, STING generally will be translocated to ERGIC or Golgi apparatus, where TBK1 is recruited to phosphorylate STING and to mediate expression of IFN, ISG, and cytokines [36]. Meanwhile, TBK1 will phosphorylate p62 and the phosphorylated p62 immediately binds to the ubiquitinated and phosphorylated STING and rapidly relocates STING to autolysosomes for degradation to terminate the innate immune response [37]. In addition, Unc-93 homolog B1 (UNC93B1) can negatively regulate STING-mediated innate immunity by promoting degradation of STING [38]. Further studies showed that UNC93B1 could interact with STING by transporting STING into autolysosomes, relying on the traffic function of UNC93B1, to degrade STING and prevent STING from over-activation [39]. Recent studies suggested that AP-1 complex may be an important destination of STING after activation, which sort and transport phosphorylated STING directly to endolysosomes for degradation through the CCV pathway [40]. Upon cGAMP-triggered translocation, ARMH3 interacted with STING at the Golgi and recruited phosphatidylinositol 4-kinase beta (PI4KB) to synthesize PI4P, which directed STING Golgi-to-endosome trafficking via PI4P-binding proteins and AP-1 and GGA2 [41]. In addition, the resting STING could maintain a biogel-like state through liquid phase separation to inhibit STING binding cGAMP and interaction with TBK1, so as to prevent STING from over-activation [42]. This liquid phase separation state may make STING more convenient for cell sorting into different vehicles by transitions to different functional states of STING in different circumstances. Our data here show that duck STING could directly interact with LC3B to induce autophagy to degrade viral proteins and achieve the purpose of inhibiting viral infection. Therefore, the entry of duck STING into the endosomes and then lysosomes in a shorter time through the autophagy pathway may be a more convenient cellular action, which plays an important role in the fight against pathogen invasion.

Upon cGAMP binding, STING departs from the ER and travels all the way through the ER-Golgi intermediate compartment (ERGIC), Golgi apparatus, TGN, endosomes, and finally to the lysosomes [9, 43]. STEEP has been recently found to interact with and mediate the STING ER departure through phosphatidylinositol 3-phosphate (PI3P) and ER curvature formation [44] where STING interact with SEC24, leading to a COPII-mediated ER-to-Golgi trafficking of STING [43]. It is conceivable that any defects that impair STING Golgi-to-endosome trafficking will lead to its retrograde transport and nullify its activation. It is equally likely that enhanced STING ER-TGN-endosomal trafficking will promote its activation or even overactivation, which may eventually accelerate degradation of STING, as we demonstrated here that overexpression of Sar1 kinase leads to STING degradation and IFN signaling downregulation (Figure 6E and F). By contrast, inhibited endosome-to-lysosome transportation enhances STING activation [9, 45]. These reports propose that host cells could transport STING into endosomes for activation or into lysosomes for degradation through different ways to precisely regulate and prevent STING from overactivation [37]. However, on the contrary, several studies and our results here protested that the STING mediated active antiviral infection autophagy plays an important and decisive role in the innate immunity by mediating the expression of IFN and ISG [9, 23, 32]. We found that when the duck STING LIR motif was mutated, it could not interact with LC3B and could neither induce autophagy nor activate IFN expression, indicating that STING activated autophagy directly affects the function of mediating innate immunity. Therefore, we propose that the LIR mediated STING interaction with LC3B may be an indispensable premise to direct activated STING moves towards endosome but the spatiotemporal regulation mechanism is unclear, which is worthy of further study in the future.

STING-mediated autophagy is highly conserved in different species, it not only resists pathogen infection [9], but also protects cells from cell death caused by H_2_O_2_ [46] and stress response caused by energy metabolism [47]. In addition to DNA virus infection activated STING-dependent autophagy [32], RNA virus infection can also induce STING-mediated autophagy [24, 48, 49]. In order to establish infection in cells, viruses often need to avoid or antagonize STING mediated autophagy. For example, both ORF10 and ORF3a proteins of SARS-COV2 can interact with STING to inhibit STING mediated antiviral autophagy [48, 49]. Migratory birds are important vectors for the transmission of viruses, such as avian influenza virus, West Nile virus, duck plague virus [25–30], the mechanism of pathogen identification and natural antiviral immunity is still enigmatic. As the model animal of waterfowl, studying and deciphering the antiviral immune mechanism of ducks is of vital importance for the control and prevention of migratory bird transmitted diseases. Our previous studies have found that duck STING can inhibit DPV infection in an IFN dependent manner [17], and in this paper, we further found that STING-mediated autophagy plays a decisive role in innate immunity mediation. DPV is an immunoinhibitory virus [29, 50], and duck plague caused by DPV infection has caused extremely serious economic losses to waterfowl industries such as ducks and geese [51, 52]. In order to establish infection in cells, DPV also needs a variety of strategies to antagonize STING mediated antiviral innate immunity. If DPV gives priority to targeting and antagonizing STING mediated autophagy, this could simultaneously inhibit the STING mediated IFN expression, so as to “kill two birds with one stone”.

In conclusion, as a model for migratory birds, we clarified here the mechanism of duck STING mediated antiviral autophagy. We found that duck STING induced autophagy needs to bind to cGAMP first and to be transported from ER to ERGIC or Golgi apparatus via the COPII complex. STING mediated autophagy can inhibit DPV virus proliferation by degrading viral proteins. Our study may draw a picture for the host resistance of diseases transmitted by migratory birds.

## Data Availability

The datasets used and/or analysed during the current study are available from the corresponding author upon reasonable request.
